# Molecular Regulation of Arbuscular Mycorrhizal Symbiosis

**DOI:** 10.3390/ijms23115960

**Published:** 2022-05-25

**Authors:** Tania Ho-Plágaro, José Manuel García-Garrido

**Affiliations:** Department of Soil Microbiology and Symbiotic Systems, Zaidín Experimental Station (EEZ), CSIC, 18008 Granada, Spain; tania.ho@eez.csic.es

**Keywords:** AM symbiosis, signal, regulators, nutrients, transcriptional regulation, autoregulation

## Abstract

Plant-microorganism interactions at the rhizosphere level have a major impact on plant growth and plant tolerance and/or resistance to biotic and abiotic stresses. Of particular importance for forestry and agricultural systems is the cooperative and mutualistic interaction between plant roots and arbuscular mycorrhizal (AM) fungi from the phylum Glomeromycotina, since about 80% of terrestrial plant species can form AM symbiosis. The interaction is tightly regulated by both partners at the cellular, molecular and genetic levels, and it is highly dependent on environmental and biological variables. Recent studies have shown how fungal signals and their corresponding host plant receptor-mediated signalling regulate AM symbiosis. Host-generated symbiotic responses have been characterized and the molecular mechanisms enabling the regulation of fungal colonization and symbiosis functionality have been investigated. This review summarizes these and other recent relevant findings focusing on the molecular players and the signalling that regulate AM symbiosis. Future progress and knowledge about the underlying mechanisms for AM symbiosis regulation will be useful to facilitate agro-biotechnological procedures to improve AM colonization and/or efficiency.

## 1. Introduction

The reciprocal relationship between microorganisms and plants has a major impact on plant ecophysiology and agricultural development. Of particular importance for forestry and agricultural systems are the beneficial interactions between plants and micro-organisms, especially in relation to rhizobacteria and rhizospheric fungi, which are capable of enhancing plant growth and tolerance and/or resistance to biotic and abiotic stresses, having particular relevance for biofertilization processes and agrobiotechnological approaches to increase plant productivity.

Arbuscular Mycorrhizal (AM) fungi belonging to the phylum Glomeromycotina are soil microorganisms that establish mutual symbiosis with the majority of higher plants. This symbiotic interaction is highly beneficial and is based in the bidirectional nutrient transfer between host plants and fungi. AM fungi provide plants with essential mineral nutrients such as phosphorus (P) and nitrogen (N) and, in return, fungi obtain their carbon from the host plant in the form of plant photosynthates and fatty acids [1,2,3]. In addition to their role in plant growth, AM fungi also enhance the ability of plants to adapt to biotic and abiotic environmental stress conditions (for review, see Pozo and Azcón-Aguilar [4] and Lenoir, et al. [5]).

**Figure 1:**
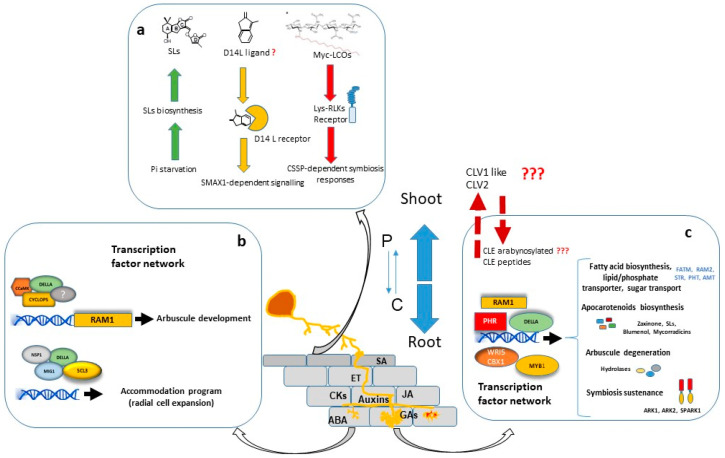
Development of the arbuscular mycorrhizal (AM) symbiosis and its molecular regulation at different stages. AM symbiosis facilitates mutual nutritional benefits: the plant provides carbon (C) to AM fungi in form of carbohydrates and fatty acids, while AM fungi provide minerals, mainly inorganic phosphate (Pi) and nitrogen to the plant. The availability of the different nutrients is a key factor determining the establishment and development of the interaction. AM symbiosis alters plant hormonal homeostasis, and phytohormones play a key regulatory role in the establishment and functioning of the AM symbiosis at different levels: regulating physiology and growth, as well as defense and stress adaptation in AM plants, and participating in the formation and turnover of AM structures. At initial stages (**a**), Strigolactones (SLs), secreted from host roots under Pi starvation, induce fungal spore germination and hyphal branching, facilitating the contact of the fungal hypha with the root and the formation of the hyphopodium. In response to AM perception, a complex of D14L receptor with a yet unknown D14L ligand is proposed to promote the degradation of SMAX1 and the downstream activation of target genes, including symbiosis-related and SL synthesis genes necessary for the accommodation of AM fungi in host cells. AM-released chitinaceous molecules (Myc-LCOs) are proposed to be detected by specific Lys-RLKs with the formation of a receptor complex that leads to the induction of the common symbiosis signaling pathway (CSSP)-dependent symbiosis responses, including transcriptional regulation of AM symbiosis-related genes, which promotes the successful colonization of host root cells by AM fungi. A number of CSSP-activated nuclear transcription factors including CCaMK and CYCLOPS form complexes with a large number of GRAS domain-containing transcription factors, including DELLA, MIG1 and SCL3 that enable the downstream activation of symbiotic genes that facilitate the morphological accommodation of the root cell hosting the fungus, and possibility arbuscule development through the activation of RAM1 (**b**). Arbuscule functionality, maintenance and turnover (**c**) are also dependent on a number of nuclear transcription factors such as PHR, RAM1, DELLA, MYB1 and WRI5A/B that regulate transcriptional programs related to biosynthesis and the transport of nutrients, and biosynthesis of apocarotenoids compounds with regulatory roles. PHRs integrate phosphate starvation signaling, regulating mechanisms for both direct uptake of soil phosphate and AM symbiosis. DELLA and MYB1 regulate arbuscule degradation through activation of genes involved in hydrolysis processes. Receptor Kinase-2 from the subfamily of land plant-specific receptor-like kinases (rice ARK1, ARK2 and SPARK1), has been characterized as having a role in the post-arbuscule formation required for sustaining root colonization. After fungal colonization, root-derived CLE peptides (MtCLE53 and MtCLE33 in *Medicago*) have been proposed as activating signals that trigger a mycorrhizal autoregulation response (AOM) in shoots. The participation of CLV1-like receptors such as SUNN and the hydroxyproline O-arabinosyltransferase RDN1 required for post-translational CLE peptide modification in the AOM signaling process has been proposed.

The creation of AM requires the host root cells to undergo significant structural and functional modifications, leading eventually to reciprocal beneficial effects. During the establishment of the symbiosis, the interaction is highly regulated by both partners at the cellular, molecular and genetic levels. Distinct steps in the development of the AM symbiosis have been characterized. The pre-symbiotic step is associated with a molecular cross-talk communication between both symbionts. Upon recognition, physical contact is established by the formation of a hyphopodium on the root surface. From here, root colonisation progresses rapidly; AM fungi invade cortical cells through a pre-penetration apparatus (PPA) and develop highly branched fungal structures termed arbuscules in inner cortical cells (Figure 1) [6]. Arbuscules are the key structures of the symbiosis and the site where the bi-directional nutrient exchange occurs. Fungal arbuscular branches are enveloped by the plant-derived periarbuscular membrane (PAM) that is essential for controlling transport and signalling processes between both partners. The AM fungal life cycle within the root is completed by the formation of storage vesicles and spores.

The establishment of AM symbiosis is precisely regulated by the host plant and the AM fungi, and it is highly dependent on environmental and biological variables [7]. Advances in investigating the mechanisms underlying molecular regulation of the AM symbiosis will facilitate our understanding about this association and can open a way to better exploit its potential applications. The present review covers the recent progress made in the molecular mechanisms and signalling that regulate AM symbiosis, focusing on nutrients, hormones and other signalling molecules with a role as regulators of AM association.

## 2. Nutritional Regulation

The main sense and the success of the AM symbiosis basically resides in the mutual nutritional benefits obtained by both interacting partners, the plant and the AM fungus. Plants provide carbon (C) to AM fungi in the form of carbohydrates and fatty acids, while AM fungi provide minerals, mainly inorganic phosphate (Pi) and nitrogen to the plants. In this context, it is logical to assume that the availability of the different nutrients is a key factor determining the establishment and development of the interaction.

Among the different exchanged nutrients, phosphate is the one that is thought to be the most important in the regulation of the AM symbiosis. Actually, high phosphate supply causes the inhibition of root colonization from a very early stage, including hyphopodia formation [8,9]. Moreover, Russo, et al. [10] found that high Pi negatively affects presymbiotic signalling, with an inhibition of the number of calcium spikes and the cells subjected to those spikes, while other studies do not support this observation [11]. Moreover, *Medicago* and rice plant mutants impaired in the mycorrhizal specific P transporter genes *MtPT4* and *OsPT11*, respectively, give place to arbuscule premature degeneration or failings in arbuscule development, and to a loss of the symbiosis [12,13]. Recent research in rice has greatly increased our understanding of the underlying molecular mechanisms involved in AM regulation by Pi starvation [14,15]. Briefly, at Pi limiting conditions, SPX proteins are degraded, allowing the PHR (Phosphate starvation response) transcription factors from the MYB family to bind to P1BS (PHR1 Binding Site) motifs present in the promoter of the so called “Pi starvation response-induced genes”, triggering the Pi starvation response (see later in more detail). AM regulation by Pi starvation is mediated to a large extent by the alteration of hormonal levels. For example, the increased contents of strigolactones are found in plants subjected to Pi deficiency conditions [16] and are recognition signals for AM fungi that promote their growth and entry into the roots [17,18]. Gibberellins are also plant hormones that mediate the regulation of AM colonization by Pi conditions [19]. In a similar manner, other phytohormones, and also miRNAs and peptide signals, may pay an essential role in the integration of the Pi status with the different stages of the AM symbiosis at local and systemic levels, as suggested by Müller and Harrison [20].

Although less studied, nitrogen is also a nutritional determinant of the interaction. While Pi flux during AMS is well studied, much less is known about the transporters involved in symbiotic nitrogen acquisition and further work is needed to determine whether the route of symbiotic N acquisition operates in plants. Nouri et al. [21] have shown that N starvation reduces the negative effect of high Pi on the AM symbiosis, and premature arbuscule degeneration observed in plants mutated for genes encoding specific mycorrhizal Pi transporters is prevented at low N conditions in the media [22,23]. The members of two protein families: AMTs and the Nitrate Transporter 1/Peptide Transporter family (NPF) have been found to be transcriptionally induced in different plant species upon AM inoculation. Moreover, suppression of arbuscule degeneration in *M. truncatula* PT4 mutants depends on AMT2;3, a symbiotic ammonium transporter [24]. Interestingly, strigolactones, the plant recognition signals that attract AM fungi, are not only delivered under low Pi conditions, but also upon N-starvation conditions [16]. Moreover, Calabrese, et al. [25] observed that in *Rhizophagus irregularis* associated with *Populus trichocarpa*, N starvation led to a general induction of fungal genes, with an overrepresentation of genes related to cell growth, membrane biogenesis, cell structural components and transport systems. On the plant side, the authors observed an induction of *Vapyrin*, suggested to be involved in cellular remodelling to support the intracellular development of fungal hyphae [26], and *DELLA*, which is required for arbuscule formation [27]. Altogether, the results of Calabrese, et al. [25] support the idea that low N conditions trigger a transcriptional regulation towards a higher fungal development. On the other hand, the increased N concentration entails a decreased mycorrhization [28]. Reciprocal C-Pi exchange during AM symbiosis is the base of the stability of the AM mutualism compared with other mutualisms [29]. In the mycorrhizal mutualism, both partners are able to preferentially reward each other, in contrast to other mutualisms in which one partner appears to have control over the other. Although we cannot discard a regulatory role of other mineral nutrients, they may have minor importance in comparison to Pi and N. For example, in petunia plants inoculated with *R. irregularis*, no AM development-sensitivity was detected for any of the alternative tested nutrients, including sulfate, calcium, magnesium, and iron [21].

Carbon availability (in form of sugars and lipids) from the plant to the AM fungus is also a factor determining AM development. In soybean plants, it has been shown that inoculation with AM fungus species exhibiting higher colonization rates, compared to those ones with lower AM colonization, is accompanied with an increased plant growth and with higher sugar contents, as well as a higher induction of genes related to sugar metabolism and transport [30]. A positive feedback might be established: AM development promotes plant growth, consequently increasing photosynthate availability, which promotes mycorrhizal development. Through overexpression of yeast invertases, Schaarschmidt, et al. [31] enhanced the levels of hexoses (the main form of carbohydrate used by the fungus) in *Medicago* roots, but they did not observe any alteration of mycorrhization. However, the increased phloem loading of photoasssimilates by overexpressing of the potato sucrose transporter SUT1 promoted AM fungal root colonization when the soil P availability was high [32]. Moreover, transgenic tobacco plants with a defective phloem loading or with a decrease acid invertase activity in roots showed a diminished mycorrhization [31]. These results show that in addition to the influence of photoassimilate levels on mycorrhizal colonization, host plants also have the ability to regulate colonization levels by regulating the flux of these sugars towards the fungus depending on other factors, such as Pi soil availability. Supporting this hypothesis, the studies of Bitterlich, et al. [33] suggest that the tomato transporter SlSUT2 uptakes sucrose from the periarbuscular apoplastic compartment back into the cytoplasm of the plant cell in order to reduce carbohydrate availability for the fungus, thereby negatively controlling fungal growth. The sugar SWEET transporters also seem to be relevant in controlling the supply of sugars to the biotrophic interface. From the 35 SWEET potato genes, 63% of them are differentially expressed upon mycorrhization, and a promoter-reporter analysis showed that three of them were expressed in arbuscule-containing cells [34]. The *Medicago* SWEET1b transporter has been shown to be involved in the transport of glucose across the peri-arbuscular membrane and to have a role in the maintaining of arbuscules [35]. The AM symbiosis regulation by sugars might also depend on the fungal transport. In this respect, it has been shown that the *R. irregularis* MST2 monosaccharide transporter is induced in arbuscules and intercellular hyphae and its silencing resulted in impaired arbuscule formation and *PT4* expression [36], suggesting that carbon and Pi exchanges are tightly linked and are required for proper arbuscule development and functioning.

Recent research has also revealed a regulatory role of lipids in AM symbiosis. The reduced lipid transfer from hosts to AM fungi inhibits arbuscule formation [37]. Notably, *Lotus japonicus* and *Medicago* mutants for the *FatM* and *RAM2* genes, which encode enzymes related with lipid biosynthesis, show a reduced AM colonization and higher ratios of collapse arbuscules [38,39,40,41,42]. The same result has been recently obtained in rice *RAM2* mutants, supporting a conserved ‘nutritional’ role of RAM2 between monocot and dicot lineages [43,44]. Furthermore, mutants for the *STR/STR2* genes, coding for two putative ABC lipid transporters, and mutants for the WRI5a transcription factor, also affect arbuscule development in *Medicago* [45,46]. Jiang et al. [46] showed that WRI5a is able to bind the *STR* and *PT4* promoters and may constitute a master regulator controlling periarbuscular membrane formation and bidirectional nutrient exchange between the two partners. Collectively, these findings indicate that lipids serve, together with sugars, as a major carbon source in plants that support and regulate fungal growth, development, and reproduction.

## 3. AM Regulation by Hormones and Other Signalling Molecules

Clear evidence shows that AM symbiosis alters plant hormonal homeostasis and almost all phytohormones have a key regulatory role in the establishment and functionality of the AM symbiosis, as shown by many studies based on the application of hormone treatments or on the analysis of plants with alterations in hormone biosynthesis or signalling. Plant hormones have been reported to act from early stages in the presymbiotic signalling to later stages, including the morphological cell root adaptations required to accommodate the hosted fungus, the control of AM colonization and the regulation of AM functionality (revised by Pozo, et al. [47], Bedini, et al. [48], and Liao, et al. [49]). Here we summarize the information obtained so far about the phytohormones involved in AM symbiosis regulation, and we also provide an overview of other identified (or hypothetical) signalling molecules from both plant and fungal origins with a regulatory role in the AM symbiosis.

Before the physical contact between the AM fungus and the plant root, a molecular communication between both symbionts is required. The key elements for this communication are strigolactones (SLs) from the plant side and the Myc factors from the fungal side. Strigolactones are a group of phytohormones derived from the apocarotenoid metabolism that regulate many aspects of plant development [50,51,52]. Concerning the role of SLs in presymbiotic signalling, once these compounds are secreted from the root to the rhizosphere in response to Pi deficiency conditions [16,53], they serve as signals indicating the presence of a host receptive to be colonized by AM fungi. SLs detection by AM induces fungal spore germination and the activation of fungal oxidative metabolism (in terms of organelle division, ATP production and gene expression) to generate the energy required to stimulate its own growth and branching, increasing the possibilities of physical contact with the host root, and preparing the fungus for the establishment of the symbiosis [17,54,55,56]. It has been observed that the promoting effect of SLs on fungal development depends on the concentrations and structural features of plant secreted SLs [57]. In addition, SLs also induce the delivery of diffusible signals by the fungus, such as short-chain chitin oligomers, which activate the common symbiosis signalling pathway (CSSP) in epidermic plant cell roots allowing initial colonization [17,50,58,59,60]. In agreement with all these observations, many reports show that mycorrhizal colonization is significantly reduced in plant mutants defective for the biosynthesis and export of strigolactones [51,53,61,62]. When the AM fungus is well stablished in the root, SLs biosynthesis is reduced, probably due to autoregulation mechanisms to avoid over-colonization once the plant has reached a better nutritional status [63].

Abscisic acid (ABA) is another apocarotenoid hormone with a regulatory role in mycorrhizal root colonization. The ABA *sitiens* tomato mutants with reduced ABA concentrations showed a reduced AM colonization and a lower percentage of well-developed arbuscules [64,65], while mycorrhizal colonization and arbuscule intensity were promoted in ABA pre-treated potato plants [66]. In vitro experiments have shown that ABA treatment induces hyphal branching around the spores, suggesting that ABA promotes fungal spore viability [66]. However, regarding the role of ABA on the fungal spore germination rate, contradictory positive [67] and negative [66] effects have been reported. Charpentier, et al. [68] showed a dual role of ABA on AM symbiosis in *Medicago*: low ABA concentrations positively regulated AM colonization with the participation of the PP2A holoenzyme subunit, while high concentration ABA treatment impaired the mycorrhizal-induced calcium spiking and negatively affected fungal colonization. These results suggest that in a first stage ABA may promote mycorrhizal development, but in sufficiently AM colonize roots ABA accumulation may reach levels with an inhibitory effect on early signalling to avoid the progression of the colonization process.

Several reports suggest that the giberellin-DELLA complex also plays an essential role in the control of symbiosis. Mycorrhizal development is ligated to the increased levels of GAs, mainly from the 13-hydrolated type, and an increased expression of genes associated to their biosynthesis [69,70,71]. Experiments with pea mutants deficient in gibberelins, as well as exogenous GA treatment applied to rice and tomato plants, support a negative regulatory role of GAs on mycorrhizal colonization [70,72,73]. In agreement with this, DELLA TFs, which are repressors of GA signalling, are required for a correct mycorrhizal colonization and arbuscule formation, as shown by research performed using pea, *M. truncatula* and rice mutants lacking or overexpressing DELLA [27,72,73]. Takeda, et al. [71] observed that exogenous GA_3_ reduced hyphal colonization and arbuscule formation in *L. japonicus*, while an inhibition of GA biosynthesis or suppression of GA signalling negatively affected fungal hyphal branching in the host roots. Similarly to the regulation of AM by ABA [68], these results also suggest a double regulatory role of GAs in the AM symbiosis: high GA concentrations may inhibit AM colonization and arbuscule formation, while certain minimum levels of GAs might be required for proper mycorrhizal development [71]. Interestingly, a recent study confirmed that GA suppresses Arum-type AM symbiosis in *L. japonicus* but, on the contrary, it shows that GAs promote Paris-type AM symbiosis in *Eustoma grandiflorum* and *Primula malacoides* [74], pointing towards a diversity of regulatory mechanisms underlying AM symbiosis among different host plants and AM morphological types.

Brassinosteroids are also thought to have a signalling role during AM symbiosis. Actually, a reduced mycorrhizal colonization is observed in both the *M. truncatula BAK1* mutant and the rice *DIMINUTO* mutants, which are impaired in the brassinoesteroid receptor and in a sterol reductase involved in brassinoesteroid biosynthesis, respectively [33,75]. Moreover, different elements related to brassinosteroid biosynthesis and signalling interact with SUT2, a sucrose transporter that negatively regulates fungal growth, probably by retrieving sugars from the symbiotic interface [33,75].

Although the role of other plant hormones in mycorrhizal establishment and development has been less studied, it is thought that most of them are involved in these processes. In this sense, several studies have suggested a role of auxin in AM initiation and also in arbuscule development [76,77]. Root auxin content was correlated with strigolactone exudation and auxin may regulate early events in the formation of arbuscular mycorrhizal symbiosis by controlling strigolactone levels [78]. After the initiation stages, auxin MiR393/AFB-dependent auxin signalling is required for AM formation, since the hampering of auxin perception via enhancing the expression of *miR393* severely impaired arbuscule development in several mycorrhizal plant species [76]. A positive correlation between the endogenous IAA content and mycorrhization level, particularly arbuscule incidence, has been demonstrated, suggesting that a mechanism to maintain cellular auxin homoeostasis is involved in finely tuning AM symbiosis [79]. In contrast, salicylic acid, ethylene and cytokinins have been reported to have a negative role on AM fungal penetration and colonization [72], while auxins have been observed to positively regulate arbuscule development and functionality [76]. For the jasmonic acid, both positive and negative effects on mycorrhization have been observed [80]. Moreover, as it is well-known, hormones do not act independently, but a complex hormonal dialogue regulates plant development and responses. In the case of AM regulation, the antagonistic interactions between ABA-ethylene and ABA-giberellin have been shown to regulate mycorrhizal development and arbuscule formation, respectively [59,65,81].

In addition to the hormonal signals, there is evidence indicating the presence of other essential signalling molecules during mycorrhization. For example, recent insights point to a role of coumarins as novel signals in the pre-symbiotic chemical dialog by promoting fungal metabolism and inducing the initial steps of AM colonization [82]. Another candidate to have a role in pre-symbiotic signalling is a hypothetical compound transported by the plant N-acetylglucosamine exporter NOPE1 (NO PERCEPTION1). This speculation is based on the studies performed by Nadal, et al. [83], who observed that the root exudates from the maize *nope1* mutant were unable to induce fungal transcriptomic responses before the physical contact and, consequently, hyphopodia formation and root penetration by fungal hyphae were impaired. In addition, recent research has shown that the KAI2/D14L and the DLK2 receptors, which are phylogenetically close to the strigolactone receptor D14, play relevant roles in the mycorrhizal symbiosis [84,85], and then it is expected that the not-yet identified corresponding ligands (of plant or fungal origin) might be important in AM signalling and regulation. D14L was identified by Gutjahr, et al. [84] as an essential protein for the AM symbiosis in rice, and required for the initiation of the AM symbiosis. The hypothetical compound able to bind and to be recognized by the D14L receptor (DWARF 14 LIKE) has been tentatively-called KAI2 ligand (KL). D14L is the homologous protein to the *Arabidopsis* KAI2 (KARRIKIN INSENSITIVE). As its name indicates, the KAI2 receptor is able to bind karrikins, which are smoke-derived butanolide molecules that stimulate seed germination after fires. In *Arabidopsis*, signal transduction of the karrikin perception has been described to operate through activation of KAI2 by MAX2 (More AXillary Growth2), an F-box protein which participates in the degradation of proteins such as SMAX1 (Suppressor of MAX2-1) [86,87,88]. Although the nature of the possible D14L-ligand triggering this response is completely unknown, the D14L-mediated symbiotic signalling pathway seems to be similar to the KAI2-signalling pathway activated by karrikins described in *Arabidopsis*. In this manner, the rice *d3* mutant (homolog to AtMAX2) is unable to be mycorrhized [62], and the rice SMAX1 has been reported as a suppressor of AM symbiosis that negatively regulates fungal colonization, as well as the transcription of symbiotic genes and the strigolactone hormone production [89]. Interestingly, KAI2 proteins are highly conserved in the plant kingdom across the evolution, and are present in plants both associated and not associated with fire-prone habitats [90,91,92]. Altogether, results obtained so far strongly suggest that the inducing role of D14L/KAI2 signalling on post-fire germination might be secondary, and most likely the main role of this pathway is related to the AM pre-symbiotic signalling. Curiously, the phylogenetically closed receptor DLK2 (DWARF14-LIKE2) was recently identified in tomato and it has also been shown to be involved in mycorrhizal regulation. However, the role of DLK2 is related to later stages of mycorrhization, acting as a negative regulator of arbuscule branching [85]. Although a possible participation of DELLA has been suggested, the signalling pathway has not yet been elucidated and, again, the possible signalling molecule triggering this DLK2-mediated repression of arbuscule development is completely unknown. Based on the high similarity between the SL receptor D14 and the D14L and DLK2 proteins, it is expected that the hypothetical symbiotic ligands of both receptors are also, as strigolactones, molecules of an apocarotenoid nature.

Apocarotenoid compounds seem to be especially important in AM regulation [93]. Apart from SLs and ABA, and the possible ligands of D14L and DLK2, other types of apocarotenoid molecules are very likely involved in the control of the AM symbiosis. In this respect, Wang, et al. [94] found that the apocarotenoid metabolite zaxinone, synthesized by the ZAS enzyme, increased during the early phase of the AM interaction and seemed to be necessary for AM formation. The authors observed that the *zas* rice mutant, with a lower root zaxinone content, displayed lower AM colonization levels and, although arbuscule morphology was not altered, the expression of symbiosis marker genes was barely detectable, suggesting that the arbuscules were not functional. Supporting the specific role of zaxinone in mycorrhization, orthologues of the rice ZAS are absent in non-AM plant species. Interestingly, the *zas* rice mutants presented higher levels of SLs, suggesting a complex cross-talk between zaxinone and SLs in the mycorrhizal symbiosis [94]. It is also worth mentioning that two other classes of apocarotenoids are well reported to be highly accumulated in mycorrhizal roots: the colorless cyclohexanone derivatives, also called α-ionol derivatives or blumenols, and the yellow mycorradicins (C14), that are assumed to give the typical yellow colouration to roots strongly colonized by AM fungi [95,96,97,98]. Treatment of blumenol C has been associated with an inhibition of fungal colonization and arbuscule formation at the early stages of AM development [99], and accumulation of the yellow pigment is correlated with arbuscule degradation [96]. By contrast, other studies have shown that reduced synthesis of blumenols and mycorradicin, due to deficiencies in the methylerythritol phosphate pathway, give way to an increased number of degenerating and senescent arbuscules [100]. Overall, data suggest that blumenols and mycorradicins might be involved in the regulation of AM symbiosis, although their specific roles are not clear.

Knowledge about the possible signals from fungal origin involved in the regulation of mycorrhization is very scarce. Due to methodological limitations to perform genetic approaches on AM fungi, it is very difficult to assign specific signalling roles to a particular fungal molecule. In this respect, a combination of two kinds of chitinaceous molecules, commonly known as Myc factors, has been shown to be essential for AM establishment [101]: the Myc-LCOs (lipochitooligosaccharides) and the Myc-COs (short-chain chitin oligomers). LysM-containing receptor-like kinases (LysM-RLKs) are part of the complexes involved in the perception of LCOs and COs. In this sense, the LCOs receptors LYK3 and NFR1 from *M. truncatula* and *L. japonicus*, respectively, are necessary for the establishment of the AM symbiotic interaction [102]. In addition, many other Lys-RLKs have been identified in the last years to be necessary for an appropriate mycorrhization and to be candidates to participate in the perception of Myc-COs or Myc-LCOs in different species. Such is the case with MaLyk1 from banana, OsCERK1 and OsLYK2 from rice, MtLyk9 from *M. truncatula*, and SlLY10 and SlLYK12 from tomato [103,104,105,106,107,108]. These reports suggest that COs and LCOs signalling mediated by LysM-RLKs is a conserved feature in AM regulation across plants.

In addition to the Myc-LCOs/COs, AM fungi also secrete proteins, known as “effectors” to communicate with the host plant and to modulate the immune response to allow mycorrhization. One of the characterized fungal effectors is SP7 (Secreted Protein7), which has been described to interact with ERP (Ethylene Response Factor) and to favour root colonization, probably by inhibiting ethylene signalling and, in this way, counteracting plant immune response [109]. Another *R. irregularis* effector is SIS1, which is induced by SLs and is necessary for the proper fungal colonization and formation of arbuscules [110]. Finally, the RiCRN1 effector has also been suggested to be essential for AM colonization and arbuscule development, as the knock- down of *RiCRN1* led to an impairment of the symbiosis in *M. truncatula*, while its overexpression led to a decrease of arbuscule size [111]. Although only SP7, SIS1 and CRN1 have been identified as fungal effectors participating in the establishment of the AM association [109,110,111], it is expected that many other effectors should be involved in AM regulation. Actually, in silico analyses in the genome of *R. proliferus* have predicted the presence of coding regions for 416 small secreted peptides [112]. Also, in the genome of *R. irregularis*, 220 candidate effector genes are present, of which 95% are also found in *R. clarus* [113]. Although this observation suggests a high conservation of the effector protein battery between AM fungal species, transcriptomic analyses showed that the expression of most of the predicted effector genes is highly dependent on the fungus, with a high variation even at an intraspecific level [114], and the expression of most of these genes also depends on the host plant species [115].

## 4. Transcriptional Regulation of AM Symbiosis

A considerable number of studies have shown that large transcriptional changes are induced in the plant host during all stages of colonization. The major portion of regulated genes is involved in signalling, protein metabolism, nutrient transport, secondary metabolite biosynthesis, cell wall modification and lipid metabolism. Furthermore, a significant number of genes encoding putative transcriptional regulators are differentially expressed in mycorrhizal roots, suggesting that AM development is regulated by a complex transcriptional control network [116,117,118,119] in which the GRAS gene family have a prominent role [120]. In agreement with this, a number of AM-responsive cis-elements in the promoter of AM-responsive genes have been identified by promoter deletion studies or computational analysis [121].

The transcriptional regulation of symbiotic genes is in part dependent on the Common Symbiosis Signalling Pathway (CSSP) activated during AM and Root Nodulation symbiosis and triggered upon perception of fungal signals. In fact, nuclear calcium oscillations generated in plant root cells upon the perception of external symbiotic signals, including Myc-LCOs, are decoded by CCaMK/DMI3, a Calcium/Calmodulin-Dependent Protein Kinase [122], which phosphorylates and activates CYCLOPS/IPD3, a primary and central transcription factor of the symbiotic signalling response [123,124]. Among other possible direct target promoters, CYCLOPS in a complex with CCaMK and DELLA binds the *RAM1* promoter and induces *RAM1* expression (Figure 1) [125], which encodes a key transcription factor required for arbuscule development. Part of the Myc-LCO and CO response is dependent on the GRAS protein NSP1 [126,127,128], probably by establishing a regulatory module together with NSP2 and the CYCLOPS-CCaMK-DELLA complex, as suggested by Jin et al. [129].

Interestingly, recent research has shown that transcriptional regulation of symbiotic genes is not only dependent on the CSSP triggered upon perception of fungal signals and mediated by CYCLOPS/IPD3, but also the phosphate starvation signalling plays a highly relevant role. In this sense, one hybrid experiment performed by Shi, et al. [14] indicates that the PHR (Phosphate starvation response) TFs govern the regulation of AM-related genes. Moreover, computational analysis carried out by these authors revealed that 42% of the promoter regions of AM-regulated genes in rice carry P1BS (PHR1 Biding Site) motifs, strongly suggesting that Pi starvation plays a central role in the transcriptional activation of a wide range of AM-symbiotic genes. Recent research in rice has greatly increased our understanding of the underlying molecular mechanisms involved in AM transcriptional regulation by Pi starvation [14,15]. Briefly, at Pi limiting conditions, SPX proteins are degraded, allowing the PHR TFs from the MYB family to bind to P1BS motifs present in the promoters of the so called “Pi starvation response-induced genes”, triggering the Pi starvation response. By contrast, at high Pi conditions, SPX proteins inhibit PHR binding to P1BS, and thus phosphate starvation–induced genes cannot be induced, inhibiting mycorrhizal infection [130].

For arbuscule development, RAM1 is assumed to be the master transcriptional regulator. *RAM1* gene expression is induced by both Pi starvation conditions through binding of PHR TF to the P1BS element of the *RAM1* promoter [14], and also by the CSSP through binding of CYCLOPS TF to a cis element (AMCYC-RE) of the *RAM1* promoter [125]. Experiments performed in *M. truncatula*, *L. japonicus* and petunia suggest that RAM1 is essential for the formation of the periarbuscular membrane and for arbuscule branching, and also probably for plant-fungus nutrient exchange [42,125,131,132,133]. Supporting these roles, roots of *ram1* mutants only show arbuscule trunks or undeveloped arbuscules [117,125,132,133], and are defective in the activation of genes involved in the formation of the periarbuscular membrane (*VAPYRIN* and *Exo70I*), in mineral transport from the fungus to the root (*PT4* and *AMT2.2*) and in fatty acid biosynthesis (*KASIII*, *DIS*, *FatM* and *RAM2*) [39,42,116,125,132,134]. Experiments performed with plants overexpressing *RAM1* validate that the activation of many of these genes related with arbuscule functionality are RAM1-dependent [125,132]. The RAM1-dependent activation of several of these symbiotic genes might be mediated by AP2 transcription factors, such as WRI5 or CBX1. Supporting this idea, experiments performed in *Medicago* indicate that RAM1 induces the expression of genes coding for specific WRI5 TFs required for arbuscule development that act as master regulators of AM symbiosis controlling lipid transfer and periarbuscular membrane formation [42,46]. Similarly, in *Lotus japonicus* the CBX1 TF is necessary for proper mycorrhizal colonization and arbuscule formation, and is also reported to activate the expression of many RAM1-dependent genes involved in lipid biosynthesis, such as *LjRAM2*, and to induce the *LjPT4* gene, with a role in Pi uptake [135]. It is worth mentioning that research performed in the monocot *Brachypodium distachyon* show slight differences regarding the relevance of RAM1 in AM regulation. Although a lower mycorrhizal colonization and a higher frequency of aberrant arbuscules was observed in *ram1* mutants of *B. distachyon*, the phenotype was milder than in dicot species, as some fully developed arbuscules were also observed [136]. This observation suggests that other proteins or pathways may compensate RAM1 function in *B. distachyon*. If this feature is general to monocots it needs to be validated in other species.

Arbuscule development needs to be accompanied by the cell expansion of cortex cells for the accommodation of arbuscules. At this regard, the adjustment of cell size during arbuscule life cycle has been suggested to be regulated by two different modules of GRAS transcription factors with antagonistic actions. On one hand, MIG1 (Mycorrhiza Induced GRAS 1) transcription factor, in a complex with DELLA, promotes radial expansion of arbuscule hosting cells while, on the other hand, MIG2 and SCL3, also in concert with DELLA, restrict cell expansion (Figure 1) [137,138].

Arbuscules are continuously being recycled. They have a relatively short life, around two to three days, and are rapidly degraded after two to seven days [139,140]. The quick removal of senescent arbuscules might be a plant regulatory mechanism to restrict the presence of fungal arbuscules that are not providing benefits to the plant. The *M. truncatula* AM-induced gene *MYB1* encodes a transcription factor which, in association with NSP1 and DELLA, has been reported as a key regulatory element required for the induction of many genes associated with arbuscule degeneration, such as cysteine proteases and chitinases [141]. Although the arbuscule lifespan is not increased in *myb1* mutants, the reduced arbuscule lifespan phenotype observed in the *mtpt4* mutant is recovered in the *pt4/myb1* double mutant. These results indicate that MYB1 accelerates arbuscule degeneration when the fungal arbuscule is not providing Pi to the plant, and suggest a functional redundancy at the MYB1 level [141]. Recent research carried out by Wang, et al. [142] in *M. truncatula* also shows that the SPX-domain containing proteins SPX1 and SPX3 regulate arbuscule degradation, probably by sensing the delivered Pi at the arbuscule level. The *spx1 spx3* double mutant has a significant lower ratio of degrading arbuscules, and exhibits a reduced expression of hydrolase genes induced by MYB1, such as Cysteine Protease 3 (CP3) and Chitinase [141,142]. The opposite phenotype has been observed in composite *M. truncatula* plants overexpressing SPX1 and SPX3 genes [142], supporting the idea that SPX1/3 play a role in regulating arbuscule degradation. Curiously, alternative roles of SPX proteins on AM regulation have been observed. As pointed out before, SPX proteins have also been reported to inhibit the rice PHR2 TF [143,144], which is involved in the transcriptional activation of Pi-starvation responsive AM-genes, and is required for proper mycorrhizal development [14,15]. Different roles of SPX proteins in AM regulation depending on the SPX family members or interactor partners, the plant species (grasses vs. legumes) and/or Pi availability and needs have been suggested [130].

Apart from the transcriptional regulation mediated by TFs, posttranscriptional mechanisms for AM gene expression regulation have been also identified. For example, miRNAs from the miR171 family seem to be involved in maintaining the balance of AM colonization [145,146]. In particular, the microRNA miR171h, which is induced in *M. truncatula* during AM colonization and by Myc-LCOs, has a repressing role on mycorrhizal colonization [145]. The underlying mechanism might be the ability of miR171h to cleave NSP2 transcripts, which encode the NSP2 TF involved in SL biosynthesis and are required for proper mycorrhizal colonization [147,148]. By contrast, *LOM1* transcripts, which also code for a GRAS TF required for root colonization, are positively regulated by another member from the miR171 family, the miR171b. In this way, miR171b stimulates AM symbiosis, probably by protecting *LOM1* transcripts from negative regulation by other miR171 members [146].

## 5. Systemic Autoregulation of AM Symbiosis

In order to balance the energy cost with the benefit gained, plants employ a systemic negative feedback loop to control the formation of nutrient-acquiring symbioses. This mechanism of feedback control is particularly important in legumes, where existing nodules systemically inhibit subsequent nodulation in other parts of the root system through a process termed “autoregulation of nodulation” (AON) [149]. A similar regulatory mechanism for AM symbiosis has been reported, and the systemic autoregulation of AM colonization (AOM) in split root studies has been observed in both legumes and non-legumes [150,151]. The AOM pathway shares some elements with AON, and physiological studies in legumes have indicated that there is at least some overlap in the genes and signals that regulate these two symbioses. This overlap is consistent with elegant studies in legumes and non-legumes, revealing that rhizobium and/or nodulation can suppress mycorrhizal development and vice versa [152,153].

Briefly, root nodule formation is controlled by a systemic feedback loop involving nodule-induced CLE peptides (CLAVATA3/EMBRYO SURROUNDING REGION (ESR)-RELATED), which in some cases appear to be arabinosylated via action of the enzyme ROOT DETERMINED NODULATION1 (RDN1), and CLV1-like leucine rich repeat (LRR) RLKs LjHAR1/MtSUNN/GmNARK in the shoots, which appears to function as a shoot receptor for root-derived CLE peptides, leading to a shoot-derived suppression of nodule numbers in the roots [154,155]. Other elements of the AON pathway include the shoot acting CLV2, CORYNE (CRN), and KLAVIER (KLV), all three of which encode LRR receptors that may also play a role in CLE perception, and TOO MUCH LOVE (TML), a root-acting F-Box protein that appears to act downstream of the shoot to root signal (revised by Wang, et al. [156]).

In addition to its role in nodulation, the CLV1-like protein is also essential in the AOM pathway, since *clv1*-like mutants across legume species (*sym29*, *sunn*, *nark*, and *har1*) also display an increased AM fungal colonization, implicating a role for these LRR-RLKs in the autoregulation of mycorrhizal symbiosis (revised by Wang, et al. [156]). Apart from the requirement of the CLV1-like protein in AOM, it is not yet clear if other AON genes encoding proteins that act in the root (RDN1, TML), shoot (CLV2, KLV, CRN) or as mobile signals (CLE) are also employed by the AOM pathway. Recently, the first genetic evidence for the AOM pathway in non-legumes has been obtained in tomato. Compared with WT, *clv2* plants displayed a significant increase in AM colonization, including arbuscule frequency, suggesting a role for the tomato CLV2 in AM development [156].

CLE peptides comprise 12–13 amino acid glycosylated peptides and work regulating developmental processes and stress responses locally and systemically [157]. The observation that Pi status and AM symbiosis regulate the expression of CLE genes in *L. japonicus* and *M. truncatula* roots [158,159,160], coupled with the studies reporting a negative regulation of mycorrhizal colonization and nitrogen response of tomato mediated by CLAVATA signalling [161], points towards a role of root-derived CLE peptide signals in AOM, probably via shoot derived SUNN-dependent signalling.

Recent studies have provided evidence for a functional role of CLE-mediated signalling in AOM [162,163]. *MtCLE53* in *M. truncatula* was induced by AM fungi and *MtCLE33* was induced in response to high Pi [162]. The overexpression of *MtCLE53* and *MtCLE33* led to significantly reduced mycorrhizal colonization compared with the control construct, and this suppression was dependent on the CLV1-like gene SUNN receptor and the hydroxyproline O-arabinosyltransferase RDN1 required for post-translational peptide modification [162,163]. Overexpression of *MtCLE53* or *MtCLE33* also led to downregulation of SL biosynthesis genes, suggesting that CLE peptides act as negative regulators of AM symbiosis, possibly via controlling the production of SLs by acting upstream of the SL biosynthetic pathway [162]. This evidence provides a new insight about the role of CLEs-SUNN in AOM through strigolactones, integrating P status and mycorrhiza autoregulation.

Intriguingly, AM fungi themselves can also produce CLE peptides that facilitate AM colonization [160] and, in addition to CLE peptides, AM symbiosis also regulates the expression of C-terminally encoded peptides (CEPs) [164] with unknown roles, opening a new avenue toward the role of small peptides and their putative leucine-rich repeat receptor-like kinases during mycorrhization.

## 6. Post Arbuscule Development Symbiosis Sustenance

AM symbioses formation and development is a highly dynamic process, where the cycle of arbuscule formation and degeneration takes only a few days. As mentioned throughout this review, the existence of molecular programmes enables the symbiotic partners to fine-tune the orchestration and coordination of the processes of recognition in the rhizosphere, fungal root invasion and cortical intracellular accommodation of arbuscules, and mechanisms that regulate AM symbiosis at different levels.

However, signalling processes that influence the balance of the interaction modulating the sustenance of the symbiosis are largely unknown. Recently, a group of rice Unknown Receptor Kinase-2 (URK2) subfamily of land plant-specific receptor-like kinases (RLKs) has been characterized having a role in post arbuscule formation [165,166]. In rice, ARK lineage proteins include ARK1, its paralogue ARK2 and the Similar Protein to ARK1 (SPARK1). ARK1 and ARK2 are both induced in arbusculated cells while SPARK1 is constitutively lowly expressed [166]. In *ark1* mutants, the fungus forms fully unfolded arbuscules but is unable to produce normal levels of vesicles and spores, correlating with an overall significantly reduced root colonisation. ARK1 is specifically expressed in arbusculated cells and localises to the periarbuscular membrane [165]. The *Ark2* rice mutant also showed normally branched arbuscules, and again showed a significantly reduced fungal root colonisation. The *ark1/ark2* double mutant reproduced the *ark1* symbiosis phenotype [166]. In conclusion, these results suggested that ARK1 and ARK2 operate in the same genetic programme acting in the same arbusculated cell, and are required for sustaining root colonisation.

## 7. Challenges and Future Perspectives

The application of powerful techniques of genetic and plant molecular biology joined with the increased interest of the scientific community for the mycorrhizal symbiosis have resulted in a rapid rise in the number of molecular studies revealing processes and mechanisms regulating AM symbiosis (Figure 1). Recent progress has been made in elucidating the underlying molecular details associated to the regulation of the symbiosis by phosphate starvation, the host recognition of Myc-CO4, the role of the D14L-signalling pathway in AM symbiosis, the participation of CLE peptides in the AOM and the existence of interconnected regulatory transcriptional modules regulating the establishment of AM symbiosis. Future research challenges will be to characterize the D14L ligand(s) and to decipher the molecular details underlying the SMAX1-dependent signalling in AM symbiosis. Mechanistic functions of PHRs and additional transcriptional networks acting downstream must be explored in the future. Whether and how CLE peptides participate to promote AOM is another open and interesting question for future studies. The hypothesis suggesting that CLE peptides act through strigolactones, integrating P status and mycorrhiza autoregulation should be demonstrated. A large set of AM-induced and AM-suppressed RLKs was identified, but only a few have been well studied. The investigation into the functions of AM-related RLKs will expand our knowledge of the ligand–receptor-mediated signalling mechanisms regulating AM symbiosis. Also, further research into the specific interactions and crosstalk between transcription factors and transcription regulators will provide a better understanding about how plants regulate the establishment of AM symbiosis. With regard to AM fungi, further research on fungal signals and effector molecules will provide knowledge about how plant cells discriminate signals and transmit the response to different microbes, how AM fungal effectors modulate the plant response, or how fungal factors influence host susceptibility and AM efficiency. Future results on these research topics will be useful to facilitate agro-biotechnological procedures to improve plant production in a more sustainable way and for the development of putatively commercially important crops with enhanced agronomic traits derived from AM symbiosis by facilitating AM colonization and/or efficiency in a specific manner.
